# Flavor-Related Quality Attributes of Ripe Tomatoes Are Not Significantly Affected Under Two Common Household Conditions

**DOI:** 10.3389/fpls.2020.00472

**Published:** 2020-05-13

**Authors:** Larissa Kanski, Marcel Naumann, Elke Pawelzik

**Affiliations:** Division of Quality of Plant Products, Department of Crop Sciences, Faculty of Agriculture, University of Göttingen, Göttingen, Germany

**Keywords:** *Solanum lycopersicum* L., aroma volatiles, flavor, post-harvest chain, household storage, quality, sensory analysis

## Abstract

Consumer complaints about the flavor of fresh tomato fruits (*Solanum lycopersicum* L.) have increased in the past few decades, and numerous studies have been done on the flavor of tomatoes and how it is influenced. However, it has not yet been taken into account how consumer handling affects the flavor when considering the complete post-harvest chain—from retailer (distributor) to retail to consumer. In this study, the impact of two household storage regimes on the volatile profile and important flavor-related compounds were examined, considering the entire post-harvest handling. New breeding lines (*n* = 2) and their parental cultivars (*n* = 3) were evaluated. Fruits were harvested ripe and stored at 12.5°C for 1 day, at 20°C for 2 days, and afterward at either 20 or 7°C for another 4 days. The aroma volatile profile was measured using GC-MS and GC-FID. A trained panel was used to characterize the sensory attributes of the fruits. In both storage regimes, the relative amount of hexanal increased during the storage period in three of the five cultivars/breeding lines while benzaldehyde was the only volatile compound that decreased significantly in four cultivars/breeding lines. The relative concentration of the precursors of lipid-derived volatiles—linoleic (C18:2) and linolenic (C18:3) acid—did not change in both storage regimes. The lycopene and β-carotene contents increased slightly during storage (20 and 7°C), as the carotenoid-derived volatile 6-methyl-5-hepten-2-one did. The fructose and glucose concentrations did not vary significantly, while the content of total soluble solids increased during both storage regimes. No significant difference could be found between the fruits stored at 20 or 7°C for 4 days after the post-harvest handling in all the parameters analyzed, including the sensory analysis, considering all cultivars/breeding lines. A storage temperature of 7°C is not detrimental for the flavor of ripe fruits under the experimental conditions used. The genetic background of the studied cultivars/breeding lines have a higher impact on the flavor variation than the two common household storage conditions when storing ripe fruits and taking the entire post-harvest handling into account.

## Introduction

Tomato (*Solanum lycopersicum* L.) is one of the most widely consumed vegetables in the world (Díaz de León-Sánchez et al., 2009), with 182 million tons produced worldwide in 2017 (FAOSTAT, 2019). However, consumers have been increasingly complaining about the flavor of the fresh fruits (Tieman et al., 2012; Klee and Tieman, 2013; Zhang et al., 2016). Reduced consumer acceptance of the fruits is caused by breeding programs that in particular focus on extended shelf-life, size, and yield in combination with inappropriate post-harvest handling conditions (Tieman et al., 2017). Nevertheless, tomato flavor is acquiring more and more importance for the consumers and, consequently, for the producers as well (Piombino et al., 2013). In this context, taste and flavor are widely used terms and very often used as synonyms. However, taste refers to the gustatory receptors on the tongue (sweet, sour, salty, bitter, and umami) while flavor is the result of taste, retronasal olfaction (perception of aroma volatiles via the mouth), and trigeminal inputs, and can be further enhanced by orthonasal olfaction (“sniffing”) (Spence, 2013, 2015). Tomato flavor is a complex interaction of aroma volatiles and taste, which is influenced also by visual and textural signals in the brain (Baldwin et al., 2008; Vogel et al., 2010). So far, over 400 different volatile organic compounds have been found in tomatoes, though only a small number contribute to the characteristic tomato flavor of the fruits (Baldwin et al., 2000; Krumbein et al., 2004). These volatiles are derived from several precursors, including fatty acids, amino acids, and carotenoids (Zanor et al., 2009; Vogel et al., 2010). Vogel et al. (2010) revealed that the reduction of apocarotenoids in fruits leads to reduced flavor acceptability and negatively correlates with sweetness perception. Other important contributors to flavor perception in tomatoes are sugars (mainly fructose and glucose) and acids (mainly citric and malic acid) (Ruiz et al., 2005; Tieman et al., 2012). However, the genetic background plays an important role in the chemical and sensory composition of the fruit (Tieman et al., 2012; Baldwin et al., 2015). The exact pathways of all aroma volatiles have not yet been identified and further studies are necessary to gain more insight in the complex interaction of aroma and the pathway of the volatiles and its precursors. The huge influence of the cultivar, affecting flavor perception was revealed by Tieman et al. (2012) as well. They observed volatile content variations with up to 3,000-fold differences across 152 studied heirloom varieties. Another study of Tieman et al. (2017) identified genetic loci and chemical compounds associated with consumer liking. They determined various flavor-associated chemical compounds of different heirloom varieties and compared them with modern cultivars. The results demonstrated that modern cultivars are not well liked, even when growing under commercial conditions and harvested fully ripe. Consequently, breeding new cultivars with enhanced flavor perception could be an approach toward flavor improvement in general, in addition to appropriate post-harvest handling (Kader, 2008). Many studies have dealt with tomato flavor (e.g., Selli et al., 2014; Klee and Tieman, 2018) and it changes during storage (e.g., Krumbein et al., 2004), especially the reduction in flavor due to the exposure of fruits to low temperatures (e.g., Maul et al., 2000; Renard et al., 2013; Ponce-Valadez et al., 2016). Household storage conditions can also affect tomato fruit quality (Renard et al., 2013). There are two commonly used handling conditions for storing tomato fruits in households, either in the refrigerator (4–8°C) or at room temperature (about 20°C). Renard et al. (2013) compared two household storage regimes that included room temperature (20°C) and refrigerated storage (4°C) for different durations. The authors reported a strong negative effect on the volatile profile of red-ripe fruits stored at 4°C, but up to 1 week (6 days) the aroma could be restored by reconditioning the tomatoes at room temperature for 24 h. In the study of Díaz de León-Sánchez et al. (2009), sensory analysis, volatiles, and alcohol dehydrogenase activity in light red tomato fruits stored at either 10 or 20°C were compared and the main changes in the aroma profile were detected after 6 days of storage, regardless of the temperature. In addition to volatile compounds, non-volatile compounds are investigated in various studies, as they are main contributors to flavor (e.g., total soluble solids, titratable acidity, carotenoids, fatty acids, and firmness) (Díaz de León-Sánchez et al., 2009; Renard et al., 2013; Selli et al., 2014). Some studies examined the postharvest storage durations of up to 3 weeks (Slimestad and Verheul, 2005; Verheul et al., 2015; Dew et al., 2016). Nevertheless, these studies only focused on parts of the postharvest process and did not consider the whole transportation route from harvest via retailing to the consumer. The demand for sustainable post-harvest handling, on the other hand, exists on both the producer and consumer sides to maintain fruit quality (Kader, 2008). New approaches have also been developed measuring taste (electronic tongue) and aroma (electronic nose) of food and beverages for objective high-throughput profiling and can be complementary to existing methods (Beullens et al., 2008). However, further research is still necessary. The objectives of this study were, therefore: (i) to compare freshly harvested fruits with fruits after short-term storage in two different household storage regimes in terms of important flavor-related quality attributes (e.g., total soluble solids, titratable acids, fructose and glucose, citric acid, volatiles, carotenoids, and fatty acids) and sensory perception, taking into account the entire transportation route, (ii) to compare two new breeding lines with their parental cultivars, focusing on the quality attributes, and (iii) to investigate the suitability of the e-tongue and determine whether the results correspond to the sensory attributes of the sensory panel evaluation.

## Materials and Methods

### Plant Material and Growth Conditions

The study was carried out at the experimental research station of the University of Göttingen, Germany (51°30′17.6′′N, 9°55′16.2′′E). Indeterminate tomato plants were grown under organic low-input conditions in the field under a shelter during summer 2018. Low-input conditions are characterized by a moderate irrigation regime and no, or only low, fertilization (European Council, 2008). Approximately 250 L/m^2^ water were irrigated throughout the complete growing season. An organic NK-fertilizer was applied once (1% concentration, Aminofert^®^ Vinasse, Beckmann & Brehm GmbH, Beckeln, Germany). Two F_4_-breeding lines (Black Cherry × Primabella and Black Cherry × Roterno F1) as well as their parental cultivars were used in this study. Black Cherry (Culinaris, Saatgut für Lebensmittel, Göttingen, Germany) and Primabella (Gärtnerei LohmannsHof, Germany) are cocktail tomatoes, while Roterno F1 (Rijk Zwaan, Netherlands) is a salad cultivar. Seeds were germinated in Bio-Traysubstrat (Klasmann-Deilmann GmbH, Geeste, Germany) under greenhouse conditions (22°C day, 18°C night, 16 h/8 h) in April 2018 and were potted 17 days after seeding in Bio-Kräutersubstrat (Klasmann-Deilmann GmbH, Geeste, Germany). After potting, plants grew at 20°C day and 15°C night (16 h/8 h) conditions in a greenhouse before planting in the field. The experimental design was completely randomized with 4 biological replications per cultivar/breeding line and 12 plants per replica, resulting in 240 plants in total. We minimized border effects by planting three plants at either end of each row and one extra row at each side of the experiment. All plants were cultivated with a distance of 0.5 m within the rows and 1 m distance between the rows.

### Harvest and Postharvest Treatments and Processing of the Samples Prior to Analyses

**FIGURE 1:**
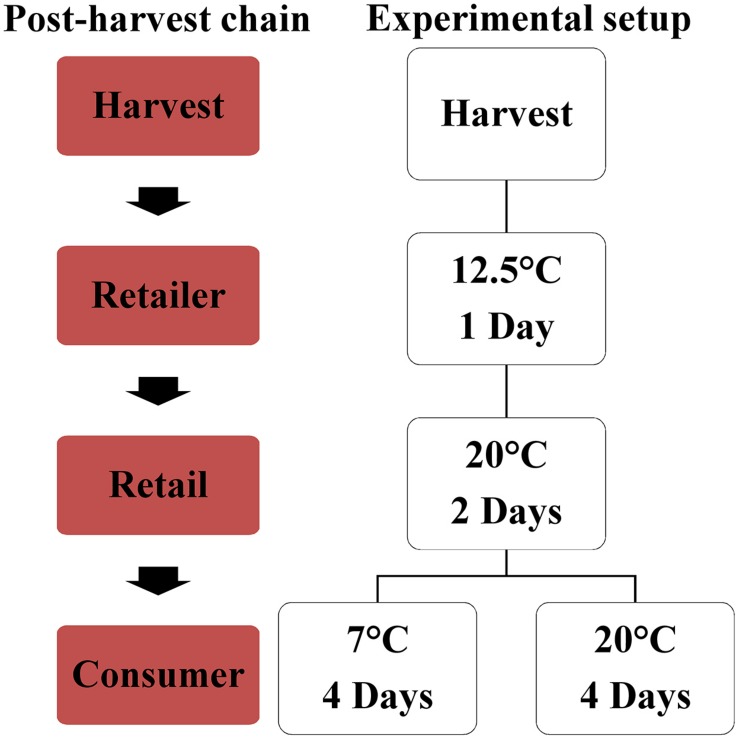
The post-harvest handling, simulating the post-harvest chain of the fruits from the harvest to the retailer (distributor), to the retail to the consumer.

For each biological replication, ripe fruits were harvested based on the color measurements at August 20th (Black Cherry, Primabella, Black Cherry × Primabella) and 22nd (Roterno F1, Black Cherry × Roterno F1) and the samples were divided for the different analyses containing the fruit quality analyses, the chemical analyses, and the sensory evaluation. The fruits were first stored for 1 day at 12.5°C (80% humidity). They were subsequently stored for 2 days at 20°C (55% humidity) and finally separated and either stored at room temperature (20°C, 55% humidity) or at refrigerator temperature (7°C, 85% humidity) for 4 days. All postharvest treatments were conducted in darkness and analyses were performed at the fruits directly after harvest as well as at the stored fruits (7 and 20°C) (Figure 1). 3 to 12 fruits per biological replication were used for each of the analyses described in the following chapters. For the color, texture, and aroma analyses as well as for panel sensory, fresh fruits were used. For the measurement of total soluble solids, dry matter, and titratable acidity, fruits were sliced, frozen and stored at −20°C until analysis. Soluble sugars, acids, minerals, and fatty acids contents were measured on freeze-dried material (freeze-dryer, EPSILON 2-40, Christ, Osterode am Harz, Germany), which was ground afterward with a ball mill. For the carotenoid and e-tongue analyses, fresh material were frozen in liquid nitrogen and subsequently stored at −20°C until analysis.

### Fruit Quality Parameters and Chemical Analyses

#### Color Measurement and Texture Analysis

The color of the fruits was measured in accordance with the CIEL^∗^a^∗^b^∗^ system [Commission Internationale de l’Éclairage (CIE), L = lightness, *a*-value = green to red, *b*-value = blue to yellow] using a Minolta Chroma meter CR-400 (Konica Minolta, Inc., Marunouchi, Japan). The hue angle [H°] was calculated according to Pék et al. (2010). Two opposite equatorial sites on each fruit were measured, with each plot containing 12 fruits. The firmness of the whole fruit was measured in Newton [N] with a texture analyzer on the equatorial site of 8 fruits (5 mm staple micro cylinder, speed: 6 mm/s, distance: 6 mm, TA.XT.plus, Stable Micro Systems Ltd, TA.XT.plus, Godalming, United Kingdom).

#### Total Soluble Solids (TSS), Dry Matter (DM), and Titratable Acidity (TA)

Frozen samples were thawed and homogenized with a hand blender for 30 s (MQ 5000 Soup, Braun, Kronberg/Taunus, Germany). 10 g of the homogenized sample were weighed in a petri dish, dried in an oven at 105°C for 1 day, and the dry matter was calculated afterward. The remaining sample was filled in 50 ml centrifuge tubes and centrifuged for 20 min at 4,696 *g* at 20°C (Centrifuge 5804 R, Eppendorf, Hamburg, Germany). A few drops of the supernatant were placed on a refractometer (handheld refractometer, A. Krüss Optronic GmbH, Hamburg, Germany) to determine TSS in °Brix. For the evaluation of TA 20 ml of deionized water and 3 ml of the supernatant were pipetted in a glass beaker and the solution was titrated with 0.1 N NaOH solution to pH 8.1 (pH-titrator Titroline 96, SCHOTT AG, Mainz, Germany). The following formula was used to calculate the titratable acidity (TA) as the percentage of citric acid, the main acid in tomato fruits:

T⁢A=m⁢i⁢l⁢l⁢i⁢e⁢q⁢u⁢i⁢v⁢a⁢l⁢e⁢n⁢t⁢o⁢f⁢c⁢i⁢t⁢r⁢i⁢c⁢a⁢c⁢i⁢d⁢(0.064⁢m⁢V⁢a⁢l)×  100usageml   0.1mol/lNaOH×  0.1mol/l×m⁢l⁢s⁢a⁢m⁢p⁢l⁢e⁢(3⁢m⁢l)(%)

#### Soluble Sugar, Acid, and Mineral Concentrations

300 mg of freeze-dried material were weighed in 15 ml centrifuge tubes and 8 ml of deionized water were added and shaken horizontally on a shaker for 1 h at room temperature. Afterward, 0.5 ml Carrez I [3.6 g K_4_Fe(CN)_6_ in 100 ml deionized water] and 0.5 ml Carrez II (7.2 g H_14_O_11_SZn in 100 ml deionized water) solutions were added, then mixed and centrifuged for 20 min at 4,696 *g* (Heraeus Megafuge 16R Centrifuge, Thermo Fisher Scientific, Waltham, United States). The supernatant was transferred in a 25 ml flask. The pellet was dissolved again in deionized water (8 ml), vortexed, and centrifuged; the supernatants were combined. In total, the procedure was performed three times and the combined supernatants were filled up to 25 ml with deionized water. The samples were filtered with filter paper (Type 615 1/4, Macherey-Nagel, Düren, Germany) in screw cap bottles and stored at −20°C until measurement. Prior to measurement, the samples were thawed, and 1 ml of the solution was filtered through a 0.45 μm PTFE syringe filter (VWR International, Radnor, PA, United States) in 1.5 ml vials. The extracts were quantified by using HPLC (Jasco, Pfungstadt, Germany) for fructose and glucose with following settings: injection volume = 20 μl; eluent = 80% acetonitrile and 20% water; flow rate = 1 ml/min; column = LiChrospher 100 NH2 (5 μm); column temperature = 22°C; refractive index detector. The settings for citric and malic acid quantification was as followed: injection volume = 10 μl; eluent = KH_2_PO_4_ (0.025 M; pH = 2.5); flow rate = 0.2 ml/min; column = ReproSil-XR 120 C8 5 μm; UV-detector = 200 nm. For the mineral concentrations, 100 mg of the freeze-dried sample were used to analyze and evaluate the minerals, as described previously in Koch et al. (2019).

#### E-Tongue

Fruits were thawed and blended with a hand blender for 30 s, filled in 50 ml centrifuge tubes and centrifuged at 4,696 *g* for 30 min at 20°C (Heraeus Megafuge 16R Centrifuge, Thermo Fisher Scientific, Waltham, United States). Afterward, the supernatant was filtered through a 615 1/4 filter (Macherey-Nagel, Düren, Germany) and stored at −80°C until analyses. A total quantity of 13.33 ml of the sample was pipetted in a glass beaker from the e-tongue and 66.67 ml deionized water were added. Subsequently, the samples were analyzed with the ASTREE Electronic Tongue (Alpha M.O.S., Toulouse, France) containing the 7-sensor array #6 (Ref. 803-0175; AHS, PKS, CTS, NMS, CPS, ANS, SCS). It is composed of a 16-position auto-sampler and a reference electrode. The inquisition time was 120 s and the cleaning time for the sensors was conducted after each measurement in deionized water for 10 s.

### Analyses of Volatiles and Important Precursors

#### Aroma Compounds

The determination of volatiles was based on the method of Olbricht et al. (2007) with some modifications. Tomatoes were washed with deionized water and cut into quarters. 50 g were weighed in 1 L beakers, 89.5 ml of a 3.18 M NaCl-solution were added, and the mixture was homogenized for 30 s with a hand blender. The mixture was filled in 50 ml centrifuge tubes and centrifuged at 4°C for 30 min at 1,690 *g* (Heraeus Megafuge 16R Centrifuge, Thermo Fisher Scientific, Waltham, MA, United States). The supernatant was transferred through a sieve and subsequently 8 ml were pipetted in a 20 ml glass vial already filled with 4 g of NaCl for saturation. 16 μl internal standard (0.16 μM 1-octanol dissolved in ethanol) was added. The sample was sealed with a magnetic crimp cap, vortexed for 10 s, and stored at −20°C until analysis. The volatiles were extracted by headspace solid-phase-micro-extraction (SPME) with a 100-μm polydimethylsiloxane (PDMS) fiber (PAL System, CTC Analytics, Zwingen, Switzerland). The incubation time before sampling was 15 min at 35°C, with an agitator speed of 500 rpm. The sample extraction time was 30 min at 35°C and in the same shaking mode. Thermal desorption in the injector was performed for 1 min at 250°C (splitless mode), followed by 9 min in split mode (split ratio 1:10). The analysis was conducted with a GC-2010 Plus (Shimadzu Deutschland GmbH, Duisburg, Germany) equipped with a flame ionization detector (FID). The FID temperature was set to 250°C. Helium was used as carrier gas with a column flow rate of 1.24 ml/min. The temperature was set to 35°C (hold for 5 min) and went up to 210°C (5°C/min). The final temperature was held for 20 min. For compound separation, a SH-Stabilwax, 0.25 mm ID × 30 m length × 0.25 μm film thickness was selected. For compound identification, a gas chromatograph coupled to a mass spectrometer (GCMS-TQ8040, Shimadzu Deutschland GmbH, Duisburg, Germany) was used with identical GC settings. Eighteen compounds were identified with NIST 14 library (National Institute of Standards and Technology, MD, United States) and confirmed with analytical standards. The mass detector was run in the electron impact ionization mode (70 eV).

#### Carotenoids Analysis

The samples were ground (30 s at 30 Hz; Retsch, model: MM 400, Haan, Germany) using liquid nitrogen for cooling. 600 mg of frozen and ground sample were weighed in 50 ml centrifuge tubes. The analysis was carried out in accordance to Serio et al. (2007) with some modifications. Instead of nitrogen flux, the *n*-hexane/carotenoids mixture was vaporized (12.45 h) using a rotary vacuum concentrator (RVC 2-25 CD plus, Christ, Osterode am Harz, Germany). Subsequently, samples were dissolved in 1250 μl ethyl acetate/dichloromethane/n-hexane (80:16:4, v:v:v) and filtrated (0.45 μm PTFE syringe filter, VWR International, Radnor, PA, United States). The samples were stored at −80°C prior to analysis with HPLC (Jasco Labor- und Datentechnik GmbH, Gross-Umstadt, Germany). A calibration curve was prepared using lycopene (CAS-Nr. 502-65-8, Roth, Karlsruhe, Germany) and ß-carotene (CAS-Nr. 7235-40-7, Roth, Karlsruhe, Germany). The carotenoids were measured with an UV-detector using spectra 454 nm for ß-carotene and 474 nm for lycopene. For the separation of the carotenoids, a Chromolith^®^ Performance RP-18e (100 × 4.6 mm) column was used. The flow rate was 1 ml/min with an oven temperature of 28°C. As mobile phase acetonitrile/water/ethyl acetate (51:7:42, v:v:v) was used and the injection volume of the sample was 10 μl.

#### Fatty Acids

The fatty acids were analyzed in respect of the method of Thies (1971) with some modifications. 350 mg of freeze-dried material were weighed in 15 ml centrifuge tubes. 2 ml of 0.5 M Na-Methylat were added and vortexed. Subsequently, 400 μl 2,2,4-Trimethylpentane and 300 μl of 5% (w/v) NaHSO_4_ were added and vortexed as well. The upper phase was pipetted in a 200 μl glass vial and stored at −20°C until analysis. For analysis, 0.6 μl of the sample was injected in the GC-FID (Thermo Electron Corporation, Trace GC Ultra; autosampler: A.L.S. 104). Injector and Detector were held at 250°C with a constant oven temperature of 205°C during analysis. Hydrogen was used as carrier gas and a Permabond FFAP-0.25 μm, 25 m × 0.25 mm column for separation. The amount of each fatty acid in the sample was expressed as a relative percentage to all determined fatty acids.

### Conventional Profiling by a Trained Sensory Panel

The sensory analyses were performed in a sensory lab with separated booths set in daylight conditions. The sensory panel consisted of 12 experienced assessors who were selected in accordance with international ISO 8586 (ISO, 2012) guidelines. The assessors were trained twice a week for 4 weeks prior to the evaluations. During the first sessions, attributes were developed to describe tomato flavor (appearance, odor, taste, and aftertaste). A list of descriptors was compiled from terms proposed by the panelists. The following sessions were used to present different references to the assessors and reach a consensus. During the training, the following eight attributes were elaborated for tomato fruits: green-grassy odor, tomato-typical odor, tomato-typical flavor, sweetness, sourness, juiciness, firmness of the fruit peel, and aftertaste. During the tests, the panelists received four quarters of tomatoes served in small bowels. Fruits stored at 7°C were adjusted to room temperature before being presented to the panelists. All fruits were cut shortly before serving to preserve the aroma. All samples were served in two replications to the panelists. The assessors evaluated the products on an unstructured line with not perceptible (0%) to highly perceptible (100%). The panelists were provided with water and unsalted cracker (P. Heumann’s Matzen, Germany) to neutralize the palate as well as coffee beans to neutralize the olfactory sense. During the test evaluation, each panelist sat in a separate booth in the sensory lab, which was designed according to the specifications of ISO 8589 (ISO, 2007).

### Statistics

Statistical analysis was performed using SPSS statistical software (IBM statistics Version 25.0, Armond, NY, United States). The results of the chemical and sensory analyses were carried out using one-way and two-way analysis of variance (ANOVA), followed by Tukey’s *Post Hoc* test (*p* ≤ 0.05). The PCA (principal component analysis) was performed for the sensory evaluation using FactoMineR package. Pearson correlations and graphs were performed within ggplot2. R version 3.6.1 was used.

## Results

### Fruit Quality Parameters

**FIGURE 2:**
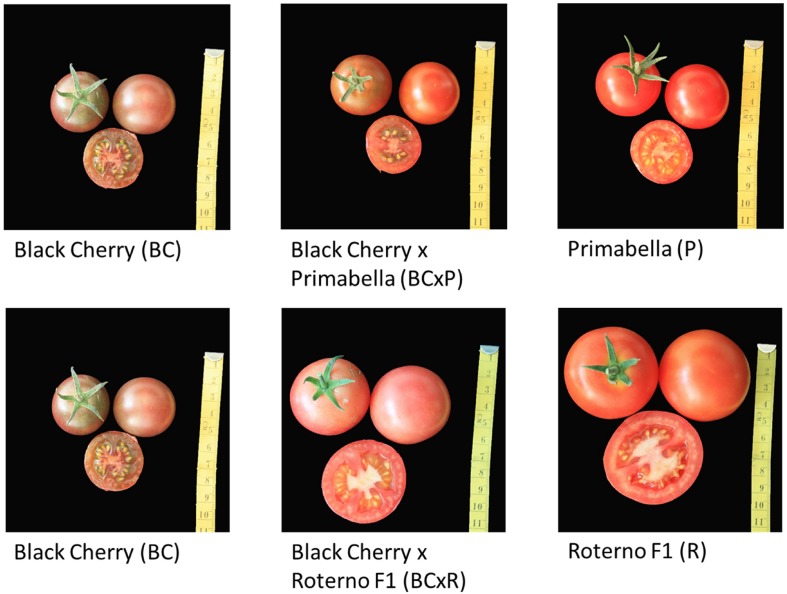
The studied breeding lines and parental cultivars.

The five cultivars/breeding lines investigated are shown in Figure 2. Black Cherry (BC) and Primabella (P) are red-brown and red cocktail cultivars, while Roterno F1 (R) is a red salad cultivar. The breeding line Black Cherry × Primabella (BCxP) has a red-brown appearance and the size of a cocktail tomato fruit. On the other hand, the breeding line Black Cherry × Roterno F1 (BCxR) is red-pink and has the size of a salad fruit (Figure 2).

**FIGURE 3:**
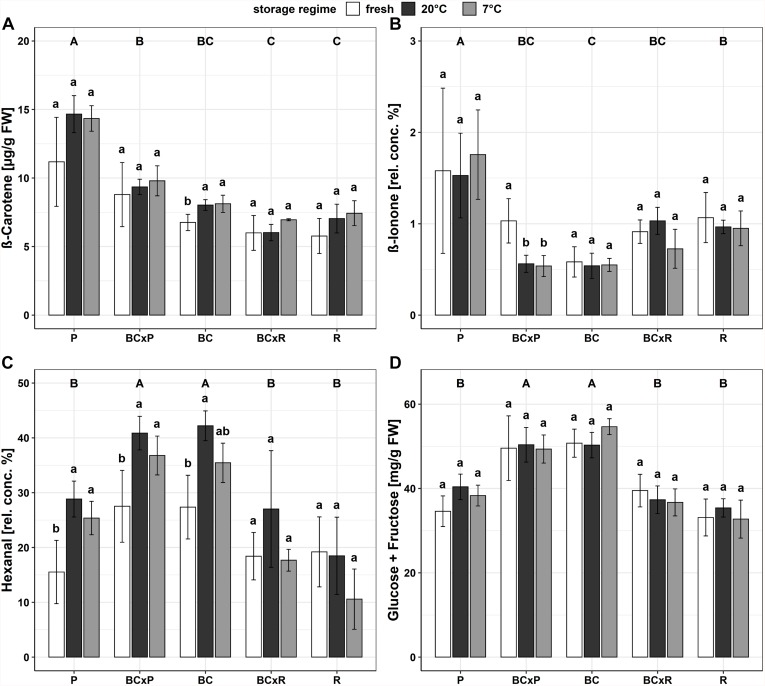
Bar plots of the different breeding lines and parental cultivars for different quality parameters, ß-carotene content **(A)**, relative concentration of ß-ionone **(B)** relative concentration of hexanal **(C**), and the sum of glucose and fructose content **(D)**. Values are means of n = 4 ± standard deviation; Tukey-Test *p* ≤ 0.05.

The cultivars/breeding lines showed significantly different contents in most quality parameters (Supplementary Table S2). With exception for cultivar R at 7°C, the lycopene and ß-carotene content increased after 20 and 7°C household storage although the difference was not significant (Table 1). The fructose and glucose concentrations did only increase significantly in one cultivar (P) after 20°C household storage (Table 1). The a-value (red color) increased significantly after the storages while the hue angle [H°] and the texture decreased significantly (Table 1). No significant change could be found in the citric and malic acid concentrations between the different post-harvest treatments (20 and 7°C) (Table 1). The fatty acid contents did not vary compared in fresh harvested fruits and fruits stored at 20 and 7°C (Table 1 and Supplementary Table S1). The magnesium and phosphorus concentrations depended on the cultivar/breeding line and were positively correlated (Supplementary Figure S1). Bar plots of important quality parameters were shown in Figure 3. They display the cultivar/breeding line and storage regime effects. The ß-carotene content was significantly the highest in P regardless of the storage conditions (Table 1 and Figure 3A). In BC, the ß-carotene content increased significantly after both storage regimes. P also showed a significant higher relative ß-ionone concentration than the other cultivars/breeding lines (Figure 3B and Supplementary Table S2). BC and BCxP contained the highest concentrations of fructose and glucose (Figure 3D). All quality parameters displayed in Table 1 showed a significant cultivar/breeding line effect, and only fructose, glucose, malic acid, potassium, phosphorus, and magnesium content did not show a significant storage effect.

**TABLE 1:** Fruit quality parameters (mean ± standard deviation) of the five cultivars/breeding lines shown for fresh harvested fruits (fresh) and after 20 and 7°C household storage with *n* = 4.

C/Bl	SR	Lycopene [μg/g FW]	ß-Carotene [μg/g FW]	Dry matter [%]	TSS [°Brix]	TA [%]	TSS/TA-Ratio	Fructose [mg/g FW]	Glucose [mg/g FW]	Citric acid [mg/g FW]	Malic acid [mg/g FW]	K [mg/g FW]	P [mg/g FW]	Mg [mg/g FW]	Fatty acid (18:2) [rel. conc. %]	Fatty acid (18:3) [rel. conc. %]	Texture [N]	a*- value	Hue-angle [°]
P	fresh	100.39 ± 5.5^a^	11.18 ± 3.2^a^	7.09 ± 0.1^b^	5.60 ± 0.2^b^	0.43 ± 0.1^a^	13.10 ± 1.3^b^	19.42 ± 2.0^b^	15.17 ± 1.7^a^	4.95 ± 1.0^a^	0.94 ± 0.2^a^	1.95 ± 0.2^a^	0.24 ± 0.0^a^	0.11 ± 0.0^a^	50.72 ± 0.5^a^	6.02 ± 0.5^a^	9.96 ± 0.4^a^	13.96 ± 0.5^b^	61.82 ± 1.1^a^
	20°C	160.45 ± 24.3^a^	14.66 ± 1.3^a^	7.79 ± 0.1^a^	6.28 ± 0.1^a^	0.37 ± 0.0^a^	17.21 ± 1.4^a^	22.75 ± 1.5^a^	17.63 ± 1.9^a^	3.89 ± 0.9^a^	1.08 ± 0.4^a^	2.12 ± 0.2^a^	0.25 ± 0.0^a^	0.11 ± 0.0^a^	50.14 ± 1.0^a^	6.05 ± 0.6^a^	5.30 ± 0.3^c^	16.74 ± 0.5^a^	55.67 ± 0.7^b^
	7°C	145.14 ± 50.3^a^	14.34 ± 0.9^a^	7.46 ± 0.3^a^	6.03 ± 0.2^a^	0.37 ± 0.0^a^	16.52 ± 1.5^a^	21.93 ± 1.3^ab^	16.37 ± 1.2^a^	4.29 ± 0.8^a^	1.01 ± 0.2^a^	1.91 ± 0.2^a^	0.19 ± 0.1^a^	0.10 ± 0.0^a^	50.65 ± 0.4^a^	6.06 ± 0.3^a^	6.14 ± 0.4^b^	15.80 ± 1.1^a^	57.13 ± 0.3^b^
BCxP	fresh	96.76 ± 23.8^b^	8.79 ± 2.3^a^	8.29 ± 0.3^b^	6.90 ± 0.3^b^	0.49 ± 0.1^a^	14.27 ± 2.1^a^	27.27 ± 4.1^a^	22.28 ± 3.5^a^	5.72 ± 0.8^a^	1.09 ± 0.2^a^	2.26 ± 0.3^a^	0.26 ± 0.0^a^	0.12 ± 0.0^a^	46.90 ± 1.2^a^	7.14 ± 0.8^a^	14.55 ± 0.3^a^	4.61 ± 1.2^b^	78.34 ± 2.9^a^
	20°C	148.73 ± 8.7^a^	9.35 ± 0.6^a^	8.80 ± 0.2^a^	7.60 ± 0.3^a^	0.47 ± 0.0^a^	16.01 ± 0.5^a^	28.13 ± 2.1^a^	22.22 ± 2.0^a^	5.00 ± 0.5^a^	1.01 ± 0.1^a^	2.39 ± 0.2^a^	0.29 ± 0.0^a^	0.13 ± 0.0^a^	47.82 ± 1.0^a^	6.17 ± 0.1^a^	8.22 ± 0.3^c^	6.96 ± 1.2^a^	71.87 ± 2.2^b^
	7°C	125.86 ± 33.1^ab^	9.79 ± 1.1^a^	8.69 ± 0.1^ab^	7.40 ± 0.2^ab^	0.45 ± 0.0^a^	16.47 ± 0.6^a^	27.51 ± 1.6^a^	21.83 ± 1.8^a^	6.54 ± 1.1^a^	1.19 ± 0.4^a^	2.31 ± 0.2^a^	0.26 ± 0.0^a^	0.12 ± 0.0^a^	48.71 ± 1.8^a^	6.36 ± 0.4^a^	10.06 ± 0.9^b^	6.15 ± 1.0^ab^	74.09 ± 2.3^ab^
BC	fresh	75.01 ± 16.1^b^	6.75 ± 0.6^b^	8.92 ± 0.2^b^	7.28 ± 0.1^b^	0.50 ± 0.0^a^	14.67 ± 0.2^c^	27.28 ± 1.9^a^	23.44 ± 1.5^a^	4.84 ± 0.3^a^	0.86 ± 0.2^a^	2.39 ± 0.1^a^	0.27 ± 0.0^a^	0.12 ± 0.0^a^	46.59 ± 0.8^a^	4.49 ± 0.3^a^	13.51 ± 0.9^a^	0.29 ± 0.4^b^	88.67 ± 1.5^a^
	20°C	116.95 ± 23.0^a^	8.03 ± 0.4^a^	9.10 ± 0.1^ab^	7.73 ± 0.1^a^	0.43 ± 0.0^b^	18.00 ± 0.3^b^	27.21 ± 1.5^a^	23.06 ± 1.6^a^	4.13 ± 0.4^a^	0.68 ± 0.1^a^	2.64 ± 0.3^a^	0.30 ± 0.0^a^	0.12 ± 0.0^a^	46.81 ± 0.8^a^	4.80 ± 0.3^a^	8.65 ± 0.7^b^	2.02 ± 0.4^a^	82.18 ± 1.5^b^
	7°C	81.00 ± 19.3^ab^	8.12 ± 0.6^a^	9.26 ± 0.1^a^	7.60 ± 0.2^a^	0.40 ± 0.0^c^	18.93 ± 0.5^a^	29.36 ± 0.9^a^	25.28 ± 1.0^a^	4.65 ± 0.8^a^	0.69 ± 0.3^a^	2.53 ± 0.2^a^	0.31 ± 0.0^a^	0.13 ± 0.0^a^	46.52 ± 0.8^a^	4.44 ± 0.2^a^	9.26 ± 0.8^b^	1.35 ± 0.4^a^	84.43 ± 1.7^b^
BCxR	Fresh	114.98 ± 17.3^a^	5.99 ± 1.3^a^	6.84 ± 0.2^a^	5.65 ± 0.2^a^	0.33 ± 0.0^a^	16.94 ± 1.3^a^	21.53 ± 2.1^a^	17.96 ± 1.8^a^	3.57 ± 0.4^a^	0.82 ± 0.1^a^	1.82 ± 0.2^a^	0.20 ± 0.0^a^	0.09 ± 0.0^a^	44.17 ± 1.0^a^	6.96 ± 0.4^a^	12.29 ± 0.5^a^	7.29 ± 1.3^b^	68.68 ± 3.4^a^
	20°C	155.25 ± 41.9^a^	6.02 ± 0.6^a^	6.41 ± 0.2^b^	5.53 ± 0.3^a^	0.28 ± 0.0^b^	19.92 ± 1.4^a^	20.87 ± 1.7^a^	16.44 ± 1.6^a^	3.08 ± 0.4^a^	1.03 ± 0.0^a^	1.78 ± 0.3^a^	0.19 ± 0.0^a^	0.09 ± 0.0^a^	45.15 ± 1.0^a^	6.34 ± 0.8^ab^	8.09 ± 0.3^b^	11.11 ± 1.2^a^	57.51 ± 2.3^b^
	7°C	126.99 ± 55.6^a^	6.95 ± 0.1^a^	6.67 ± 0.2^ab^	5.58 ± 0.3^a^	0.32 ± 0.0^ab^	17.77 ± 2.1^a^	20.38 ± 2.2^a^	16.31 ± 1.1^a^	4.20 ± 0.9^a^	1.03 ± 0.3^a^	1.84 ± 0.2^a^	0.20 ± 0.0^a^	0.09 ± 0.0^a^	44.68 ± 1.0^a^	5.87 ± 0.3^b^	8.87 ± 1.4^b^	10.18 ± 1.0^a^	59.88 ± 1.6^b^
R	Fresh	106.26 ± 14.0^a^	5.77 ± 1.3^a^	5.90 ± 0.2^b^	5.10 ± 0.1^b^	0.30 ± 0.0^a^	17.00 ± 1.3^a^	17.69 ± 2.4^a^	15.43 ± 2.0^a^	3.89 ± 1.3^a^	1.33 ± 0.4^a^	1.65 ± 0.1^a^	0.19 ± 0.0^a^	0.09 ± 0.0^a^	46.25 ± 1.3^b^	6.77 ± 1.5^a^	18.54 ± 0.8^a^	15.85 ± 0.4^b^	61.27 ± 0.5^a^
	20°C	127.92 ± 26.0^a^	7.04 ± 1.0^a^	6.58 ± 0.1^a^	5.48 ± 0.2^a^	0.31 ± 0.0^a^	17.71 ± 1.1^a^	19.37 ± 1.0^a^	16.01 ± 1.3^a^	3.52 ± 0.4^a^	1.18 ± 0.3^a^	1.85 ± 0.2^a^	0.20 ± 0.0^a^	0.10 ± 0.0^a^	48.69 ± 1.2^a^	5.85 ± 0.7^a^	12.06 ± 1.0^b^	17.55 ± 0.8^a^	56.93 ± 0.9^b^
	7°C	105.94 ± 9.9^a^	7.43 ± 0.9^a^	6.50 ± 0.3^a^	5.20 ± 0.2^ab^	0.28 ± 0.0^a^	18.57 ± 1.6^a^	17.70 ± 2.4^a^	15.03 ± 2.1^a^	4.47 ± 1.5^a^	1.65 ± 0.7^a^	1.67 ± 0.1^a^	0.20 ± 0.0^a^	0.09 ± 0.0^a^	47.98 ± 0.7^ab^	5.72 ± 0.2^a^	12.60 ± 1.0^b^	17.44 ± 0.8^a^	58.04 ± 1.5^b^
C/Bl		**	***	***	***	***	***	***	***	***	***	***	***	***	***	***	***	***	***
SR		***	**	***	***	***	***	ns	ns	**	ns	ns	ns	ns	*	**	***	***	***
C/Bl × SR		ns	ns	***	*	*	*	ns	ns	ns	ns	ns	ns	ns	ns	ns	*	ns	*

Different letters indicate significant differences between fresh harvested fruits, after 20 and 7°C household storage for each cultivar/breeding line (Tukey-Test *p* ≤ 0.05). TSS, total soluble solids; TA, titratable acidity; C, cultivar; Bl, breeding line; SR, storage regime; K, potassium; P, phosphorus; Mg, magnesium. ns, not significant, *p < 0.05, **p < 0.01, ***p < 0.001.

### Volatiles and Important Precursors

The volatile profile was analyzed, identifying 18 different aroma compounds considered to contribute to the tomato flavor in fruits, as shown in Table 2. Significant changes of the aroma compounds were always found in single cultivars/breeding lines, depending on the cultivar/breeding line and the compound, but not in all cultivars/breeding lines (Supplementary Table S1). The aroma compounds hexanal, 6-methyl-5-hepten-2-one, (Z)-3-hexenol, 2-isobutylthiazole, benzaldehyde, and (E)-geranylacetone showed both cultivar/breeding line and storage effects (Supplementary Table S1). Benzaldehyde was the only aroma compound, which decreased in all cultivars/breeding lines after both household storage treatments (20 and 7°C) (Table 2). Nevertheless, a significant reduction could only been seen in P, BC, and BCxR. Hexanal increased significantly after both treatments, except in R, BCxR and BC at 7°C (Table 2 and Figure 3C). The fatty acids-derived volatiles (*Z*)-3-hexenal and (*E*)-2-hexenal correlated positively (Supplementary Table S4), but the behavior during storage depended on the cultivar/breeding line as well (Table 2). The relative concentrations of the two aroma volatiles increased in BCxP but decreased in the other cultivars/breeding lines compared with the fresh fruits, except in BCxR at 7°C, where they also increased (Table 2). The relative 2-isobutylthiazole concentration was significantly the highest in the cultivar R (Supplementary Table S1). The relative concentration of the carotenoid-derived volatile 6-methyl-5-hepten-2-one increased after harvest at both storage conditions (20 and 7°C) (Table 2). 6-methyl-5-hepten-2-one showed a significantly positive correlation with lycopene (Supplementary Table S3), but not with ß-carotene (Supplementary Table S3). On the other hand, ß-ionone was positively correlated with ß-carotene but not with lycopene (Supplementary Table S3).

**TABLE 2:** Eighteen aroma compounds in tomato fruits and their relative fold changes after storage at 20 and 7°C compared to fresh harvested fruits are shown.

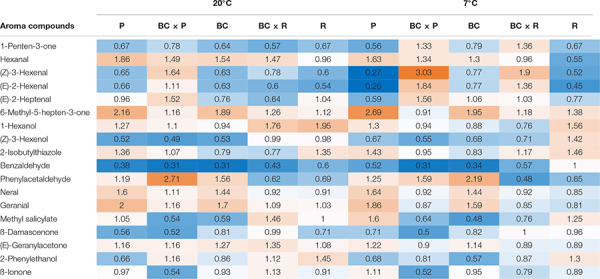

*In total, 71 detected aroma compounds were normalized to 100% and the relative concentrations were used for the calculation of the relative fold changes (FC) in each aroma compound (*n* = 4); the color scale ranges from 3 (orange) to 0.2 (dark blue) and corresponds to the FC.*

### Sensory Evaluation

**FIGURE 4:**
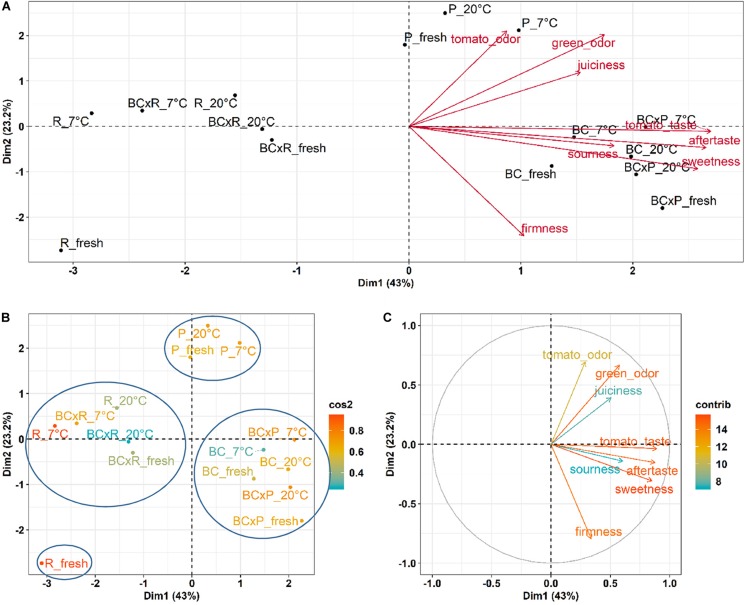
PCA results for the sensory evaluation of the different cultivars/breeding lines compared fresh fruits and fruits stored at 20 and 7°C. PCA show the cultivars/breeding lines and the explanatory variables **(A)**, the quality of representation (cos2) of the cultivars/breeding lines on the two dimensions **(B)**, and the contribution of the sensory variables to the principal components in percentage **(C)**.

To show the relations between the cultivars/breeding lines and the different storage conditions, we performed a Principal Component Analysis (PCA) (Figure 4).

**FIGURE 5:**
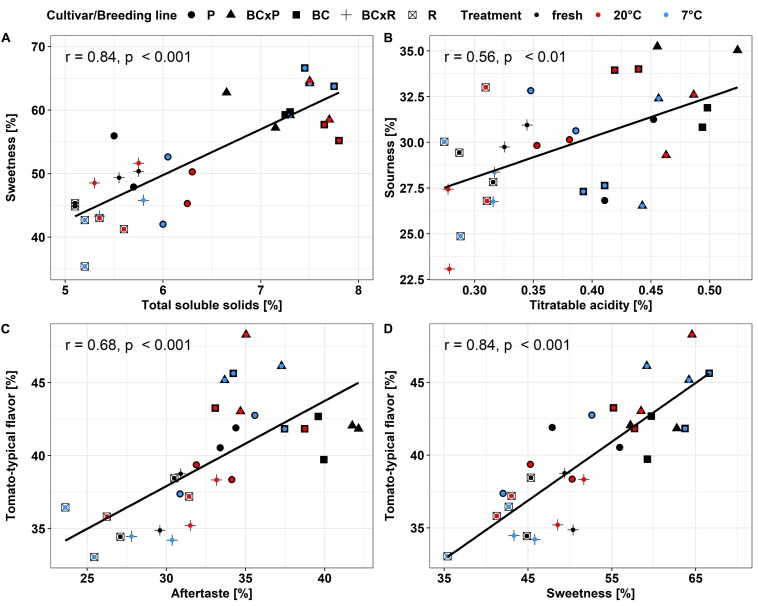
Pearson-correlation of the evaluated sensory attributes, total soluble solids and sweetness **(A)**, titratable acidity and sourness **(B)**, aftertaste and tomato-typical flavor **(C)** and sweetness and tomato-typical flavor **(D)** in the different breeding lines and parental cultivars. Values are means of 10–12 panelists; correlations were significant with *p* < 0.05.

**FIGURE 6:**
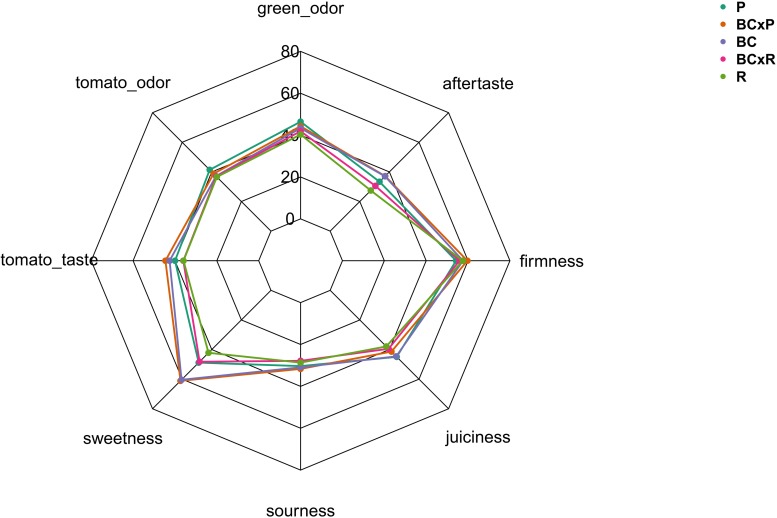
Sensory evaluation of five different breeding lines/cultivars. Evaluation was conducted with 10–12 panelists.

The biplot in Figure 4A illustrates the cultivars/breeding lines and explanatory variables. BC and BCxP correlated positively with Dimension 1 (Dim1), regardless of whether the fruits were stored at 20 or 7°C. R and BCxR correlated with the negative values of Dim1, while P correlated with the positive values of Dimension 2 (Dim2) (Figure 4A). We could separate the cultivars/breeding lines into four groups (Figure 4B). BC_fresh, BC_7°C, BC_20°C, BCxP_fresh, BCxP_7°C and BCxP_20°C are relatively close together. The same becomes clear for the samples BCxR_fresh, BCxR_7°C, BCxR 20°C, R_7°C, and R_20°C on the opposite side of the first dimension. R_fresh, on the other hand, stands more by itself, and the last group consists of P_fresh, P_7°C, and P_20°C (Figure 4B). The cultivars/breeding lines that are on the same side of the given variable have a high value for those variables. Figure 4C shows the plotted sensory variables, while the different color shades represent the contribution of the variables in percentage terms to the principal components. Dim1 positively correlates with sweetness, aftertaste, tomato_taste, firmness, green_odor, and sourness. Tomato_odor is the variable more represented on Dim2 and correlated positively with its values (Figure 4C). The quality of representation of the cultivars and the breeding lines are plotted in Figure 4B. A high cos2 (square cosine) indicates a good representation of the individuals on the principal components. A comparison of sweetness and sourness with laboratory analyses showed significant correlations between the results of the human senses and the instrumental measurements (Figures 5A,B). The sensory analyses did not reveal significant storage effects but cultivar/breeding line effects (Supplementary Table S5 and Supplementary Figure S2). BC and BCxP were significantly higher rated in sweetness, regardless of the post-harvest conditions (Figures 5A, 6). Measured sweetness and aftertaste by the panelists showed a positive correlation with the tomato-typical flavor (Figures 5C,D). BC and BCxR were rated significantly highest in tomato-like flavor (Figure 6) and R and BCxR were rated the lowest in the attribute aftertaste in the panel evaluation (Figure 6).

### Sensory Evaluation and E-Tongue Results

**FIGURE 7:**
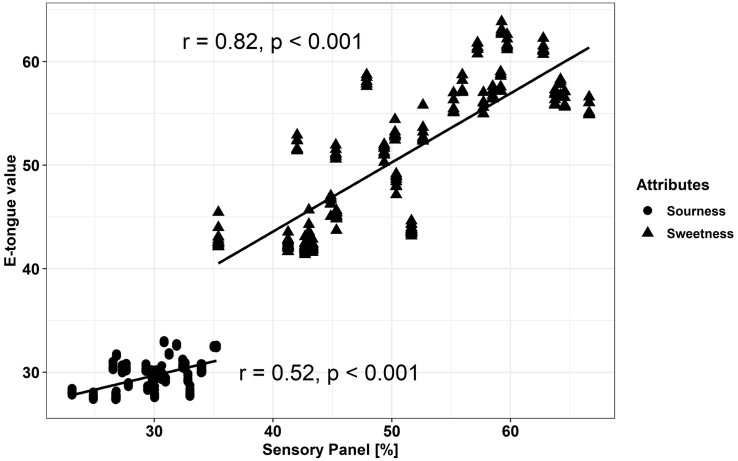
Comparison of sensory measured results and e-tongue values for sweetness and sourness (Pearson-correlation) within fresh fruits and fruits stored at either 20°C or 7°C. E-tongue results are mapped with six out of seven sensors (without sensor AHS). Results of fresh fruits, after 20 and 7°C storage are included in the correlation; correlations were significant with *p* < 0.05.

The electronic tongue (e-tongue) has been applied to measure taste with regard to the five basic tastes of the human senses (sweetness, sourness, bitterness, saltiness, and umami). We compared the output of the e-tongue results for sweetness and sourness to the sweetness and sourness perception of a trained sensory panel. The results from the sensory panel and the measured data from the electronic tongue showed a significant positive correlation for sweetness (*r* = 0.82) and sourness (*r* = 0.52) (Figure 7).

## Discussion

In the present study, the entire transportation route of tomatoes from harvest via retailer (distributor) to retail to the consumer was evaluated. We focused on the short-term fruit storage, considering the entire post-harvest chain and studied the impact of two typical household storage conditions (20 and 7°C) in this context. Harvesting in practice and following a typical post-harvest chain, which includes one day at the distributor (12.5°C) and two days at the retail (20°C) before reaching the consumer. Two new breeding lines and their parental cultivars were evaluated, because little is still known about the fruit flavor behavior when fruits are harvested ripe and undergo the whole transportation route.

### Influence of Storage Conditions on Important Fruit Quality Parameters of New Breeding Lines and Their Parental Cultivars

The influence of the cultivars/breeding lines was higher than that of the different postharvest handling during simulated household storage. We found high correlations between the measured total soluble solids and the analyzed fructose and glucose concentrations in the fruits with regard to all cultivars/breeding lines as well as significant correlations between titratable acidity as well as citric acid and malic acid (data not shown). These results also clearly showed the variation in these compounds with regard to the cultivars/breeding lines and the importance of these taste-related compounds, which are well-shown in various studies (e.g., Malundo et al., 1995; Piombino et al., 2013; Baldwin et al., 2015). The dry matter content positively correlated with total soluble solids and these were positively correlated to fructose and glucose concentrations and the sweetness perception recorded by the trained panel (data not shown). Enhancing the dry matter content of the fruits could be an interesting approach toward flavor enhancement. Tieman et al. (2017) showed in their study that the negative correlation of fruit weight and sugar content could be linked to the reduction of the high-sugar alleles caused by enhancing the fruit size during breeding. Consistent with our study, Zhang et al. (2016) found no alteration in sugar or acid concentrations after cold storage (5°C). In the present study, the total soluble solids content and the *a*-value (red color) tend to be higher in the stored fruits compared to the fruits analyzed directly after harvest. These results are consistent with Verheul et al. (2015). We found differences in the analyzed parameters between fresh and stored fruits, but no difference between the two short-term household storage regimes, when all fruits pass the same transport route before being stored by the consumer at different temperatures – e.g., at room temperature or in the refrigerator.

### Volatiles and Important Precursors

Important volatiles contributing to the tomato flavor of fruits are derived from carotenoids and fatty acids (Farneti et al., 2015) though the pathway of the exact biosynthesis of all aroma volatiles has not been clarified yet (Mathieu et al., 2009). The carotenoid-derived volatiles are produced by cleavage of the carotenoids present in the fruits, whereas the most abundant carotenoids are lycopene and ß-carotene (Lewinsohn et al., 2005; Klee and Tieman, 2018). In our study, we analyzed these main carotenoids, which are precursors of ß-ionone, geranylacetone, geranial, and 6-methyl-5-hepten-2-one (Farneti et al., 2015). Apocarotenoid volatiles can be separated into linear apocarotenoids—such as geranylacetone and 6-methyl-5-hepten-2-one—and cyclic apocarotenoids—namely ß-ionone—and are positively linked to flavor acceptability, having fruity and floral perceptions (Vogel et al., 2010). We found the highest concentrations of both ß-carotene and ß-ionone in the cultivar P. That can be explained by the observation that apocarotenoid volatiles and carotenoid precursors are shown to be proportional to each other (Vogel et al., 2010). The volatile ß-ionone is a direct breakdown product of ß-carotene (Lewinsohn et al., 2005) and so, is related to it. Our results are consistent with studies from Baldwin et al. (2000) and Vogel et al. (2010). With respect to the entire transportation route in our study, most carotenoid-derived volatiles increased slightly during both household conditions viz refrigeration (7°C) and room temperature (20°). A significant increase was only found for the volatile 6-methyl-5-hepten-2-one and only in cultivar P, compared to its content in fresh fruits analyzed directly after harvest. These results were similar with the results from Farneti et al. (2015), who outlined that the carotenoid-derived aroma compounds, e.g., geranylacetone and ß-ionone, responded less severely and were in red ripe tomatoes more cultivar-dependent during cold storage conditions. During the storage at 16°C, these volatiles increased, while they constantly decreased during 4°C and Farneti et al. (2015) discussed the observed accumulation of carotenoid-derived volatiles during 16°C storage as a consequence of postharvest ripening. Our results did not show a significant increase in lycopene and ß-carotene content but tend to increase. We found a significant higher coloration (increased *a*-value), which is directly linked to the lycopene content in the fruits (Arias et al., 2000). Nevertheless, we could not find significant differences in the relative content of 6-methyl-5-hepten-2-one, geranial, ß-ionone, and ß-damascenone in the cultivars/breeding lines after both storage regimes. In general, the behavior of the studied aroma compounds was not consistent during the two household storages, with one possible reason being that the cultivars/breeding lines respond differently to the treatments. Another important group of precursors are fatty acids. Whereas, the fatty acid-derived volatiles are formed during cleavage of linoleic (C18:2) and linolenic acid (C18:3) by lipoxygenase, which catalyzes the first step of the fatty acid degradation (Renard et al., 2013). This pathway is the origin of the C6 volatiles in tomato fruits, that include e.g., (*Z*)-3-hexenal, (*E*)-2-hexenal, hexanal (Tieman et al., 2012; Renard et al., 2013; Klee and Tieman, 2018). Hexanal is the most abundant volatile in tomato fruits and has been described as “green, grassy” (Ruiz et al., 2005). During our storage study with five cultivars/breeding lines, we found a significant increase in the relative amount of hexanal in the fruits of P, BCxP after either 20 or 7°C household storage and in the fruits of BC at 20°C. In the fruits of BCxR and R no significant change could be observed, which might be a cultivar/breeding line effect. (*Z*)-3-hexenal and (*E*)-2-hexenal did not vary significantly after both storage regimes. Renard et al. (2013) observed an increase in hexanal as well during the 20°C storage regime of red-ripe tomatoes, while (*Z*)-3-hexenal and (*E*)-2-hexenal did not change. In contrast to our study, hexanal decreased during cold storage (4°C) (Renard et al., 2013). In the study of Farneti et al. (2015), commercially grown red ripe tomatoes were stored at 16°C up to 20 days. The level of hexanal thereby did not show a significant change but (*E*)-2-hexenal constantly decreased during the storage period. In a study from Ruiz et al. (2005) with one commercial and four traditional varieties they observed that the variety with the highest hexanal and (*Z*)-3-hexenal content got the highest rankings for “flavor” and “overall acceptability” during a sensory test with untrained tasters. In an additional study by Baldwin et al. (2015), hexanal enhanced the overall flavor in combination with the sweet/sour, TSS/TA ratio. In the present study, the highest rated cultivars/breeding lines in sweetness and tomato-like flavor also contained the highest relative amount of hexanal. The fatty acid contents from the fresh and stored fruits (20 and 7°C), on the other hand, did not show a notable shift in the composition, whereas another study from Renard et al. (2013) observed an increase in the linoleic (C18:2) and linolenic (C18:3) acid concentrations during storage at 4°C. Our results indicate that the behavior of the fruit during cold storage (7°C) is also strongly dependent on the cultivar/breeding line. For example, the up- or down-regulation and restoration of volatiles, namely carotenoid or fatty acid-derived volatiles, underlines the great impact of the cultivar on the flavor of the fruits and the acceptance by the consumer. We did not observe the severe negative effect of cold storage compared to some other studies, which could be linked to the studied cultivars/breeding lines as well as to the chosen short-term storage regime and the fact that the fruits were harvested ripe.

### Sensory Evaluation of the Breeding Lines and Their Parental Cultivars

In our study, the results from instrumental analyses of total soluble solids and titratable acidity, reflecting the sugar and acid content of tomato fruits, showed high correlations to the sweetness and sourness perception of the fruits elevated by the sensory panel. These results are confirmed by Baldwin et al. (2015), who analyzed 38 tomato genotypes over 7 years. Their study also emphasized that higher sweetness and sugar contents correlated positively to overall flavor, which is comparable to our data within the positive correlation of sweetness and tomato-typical flavor ratings. Principal component analyses of the five studied cultivars/breeding lines analyzed directly after harvesting ripe fruits, as well as stored at room temperature or in cold storage, following the post-harvest chain, showed the discrimination between the cultivars/breeding lines. The results from the sensory evaluation showed no significant differences between the two storage regimes as well as between stored and fresh fruits. In contrast to the cultivars and breeding lines that were significantly different. Krumbein et al. (2004), who investigated the effects of a household condition (20°C) on aroma and sensory attributes of three different tomato cultivars up to 21 days, found changes in the aroma volatiles, namely an increase in hexanal and 2-isobutylthiazole, and in the investigated sensory attributes, including odor, flavor, and aftertaste. In contrast, Auerswald et al. (1999) revealed no change in the characteristic flavor, mouthfeel, and aftertaste after four and seven days at 20°C in a consumer evaluation, which showed that differences were not perceived from the human senses. A consumer panel test, which evaluated fruits refrigerated at 5°C for seven days, followed by a one day recovery period at 20°C, showed significant lower ratings in the overall liking and illustrated the adverse impact of cold storage (Zhang et al., 2016). Furthermore, the fruits analyzed in the aforementioned study were evaluated already after one and three days of cold treatment as well, which showed no significant effect in loss of volatile compounds. This is comparable to our observed results. We could not find significant differences between the two post-harvest conditions in respect of their sensory attributes, but differences between the cultivars/breeding lines. The variation is visualized in the PCA, showing that the cultivar R and the breeding line BCxR were less associated with the attributes sweetness, tomato-like flavor, and aftertaste and the third group with regard to P was more associated with the attributes green/grassy odor and tomato-like odor. The results of the fruits from the R_fresh deviate from this. They were rated differently compared to the stored fruits of this cultivar. This could be caused by after-ripening effects. Therefore, cultivars with improved flavor composition are a target for breeders, as the strong impact of the cultivar on flavor could be outlined in the present study.

### Comparison of Sensory Evaluation and E-Tongue Results

The electronic tongue (e-tongue) is used to evaluate the five basic tastes—sweet, sour, salty, bitter, and umami—in food and beverages and meant to mimic taste perceptions of humans (Baldwin et al., 2011; Xu et al., 2018). Xu et al. (2018) evaluated four different tomato cultivars at six maturity stages and after refrigeration and blanching, looking at the possibilities of discrimination via the e-tongue. The utilized sensor set comprised the following sensors: ZZ, JE, BB, CA, GA, HA, and JB and they successfully predicted the TSS levels in tomatoes. However, with regard to the correlation to TA, the sensors seemed less reliable. We found both, significant correlations for the sensory attributes sweetness and sourness, obtained with a trained sensory panel, compared to the e-tongue results. Nevertheless, the strength of the correlation with the e-tongue sensors was stronger for sweetness than for sourness. Beullens et al. (2008) predicted individual taste compounds (glucose, fructose, citric acid, malic acid, glutamic acid, sodium, and potassium), which did not show satisfactory results, except for glutamic acid and sodium, while the correlations for the tomato taste-related attributes to sensory panel evaluation showed a better result. The results in the present study show that the classification of the tested tomato cultivars/breeding lines and the prediction of tomato taste of at least sweetness and sourness is possible, which was also revealed in similar studies (Beullens et al., 2008; Baldwin et al., 2011). The e-tongue, therefore, could be an interesting tool for the evaluation and discrimination of these two important quality attributes in tomato fruits.

In summary, considering the numerous, diverse discussions about tomato flavor, we see the difficulty of this complex topic and that many factors influence this sensitive quality parameter. Taking the whole transportation route into account, the difference between fruits stored for four days at 20 or 7°C during household storage does not have a notably influence on the human perception when fruits were harvested ripe. We showed that flavor is severely dependent on the cultivar and that crossing cultivars with enhanced flavor perception is a valuable step to improve flavor perception. The next step is to look on the entire transportation route from the producer to the consumer, finding a way to preserve the flavor of the tomato fruits. We could show that harvesting ripe fruits and storing them only for a short duration, even at 7°C, can preserve tomato flavor. The e-tongue could be used to generate taste contributors and function as a supporter for flavor improvement.

## Data Availability Statement

All datasets generated for this study are included in the article/Supplementary Material.

## Ethics Statement

The studies involving human participants were reviewed and approved by Ethikkommission, University of Göttingen, P.O. Box 37 44, 37027 Göttingen, Chair: Prof. Dr. Hans Michael Heinig, Office: Dr. Michael Müller Bahns, Research Department. The patients/participants provided their written informed consent to participate in this study.

## Author Contributions

LK, MN, and EP planned and designed the experimental setup and wrote the manuscript. LK performed the experiments and analyzed the data.

## Conflict of Interest

The authors declare that the research was conducted in the absence of any commercial or financial relationships that could be construed as a potential conflict of interest.
