# Supplementary material for: Flavor-Related Quality Attributes of Ripe Tomatoes Are Not Significantly Affected Under Two Common Household Conditions
Source: Front Plant Sci. 2020 May 13;11:472. doi: 10.3389/fpls.2020.00472 (PMC7237721; doi:10.3389/fpls.2020.00472)

Supplementary Material

# Supplementary Figures and Tables

**Table S1.** Fruit quality parameters (mean ± standard deviation) of the five cultivars/breeding lines shown for fresh harvested fruits (fresh) and after 20°C and 7°C household storage with n=4. Different letters indicate significant differences between fresh harvested fruits, after 20°C and 7°C household storage for each cultivar/breeding line (Tukey-Test p ≤ 0.05). MHO = 6-Methyl-5-hepten-3-one, C = Cultivar, Bl = Breeding line, SR = Storage regime.

| **C/Bl** | **SR** | **Chroma** | **16:0**  **[rel. conc. %]** | **18:0**  **[rel. conc. %]** | **18:1**  **[rel. conc. %]** | **20:0**  **[rel. conc. %]** | **1-Penten-3-one**  **[rel. conc. %]** | **Hexanal**  **[rel. conc. %]** | **(Z)-3-Hex**  **enal**  **[rel. conc. %]** | **(E)-2-Hexenal**  **[rel. conc. %]** | **(E)-2-Heptenal**  **[rel. conc. %]** | **MHO**  **[rel. conc. %]** | **1-Hexanol**  **[rel. conc. %]** |
| --- | --- | --- | --- | --- | --- | --- | --- | --- | --- | --- | --- | --- | --- |
| **P** | **fresh** | 29.75±1.1a | 15.34±0.3a | 5.63±0.1a | 20.95±0.5a | 0.76±0.0a | 0.09±0.1a | 15.53±5.8b | 8.47±8.2a | 11.62±9.4a | 0.27±0.1a | 3.45±1.2b | 0.56±0.2a |
|  | **20°C** | 29.65±0.9a | 16.24±0.8a | 5.67±0.6a | 20.63±0.4a | 0.77±0.1a | 0.06±0.0a | 28.83±3.3a | 5.51±3.9a | 7.65±5.7a | 0.26±0.2a | 7.45±3.7ab | 0.71±0.2a |
|  | **7°C** | 29.10±2.0a | 15.85±0.5a | 5.28±0.2a | 21.05±0.3a | 0.72±0.0a | 0.05±0.0a | 25.37±3.0a | 2.26±0.3a | 3.07±0.8a | 0.16±0.0a | 9.27±1.1a | 0.73±0.3a |
| **BCxP** | **fresh** | 22.55±1.0a | 18.26±0.9a | 5.59±0.2a | 20.66±1.3a | 0.85±0.0a | 0.09±0.1a | 27.51±6.6b | 2.60±1.7a | 6.09±6.6a | 0.27±0.1a | 7.29±2.0a | 0.48±0.0a |
|  | **20°C** | 21.95±1.1a | 17.71±0.1a | 5.65±0.2a | 21.03±0.9a | 0.87±0.1a | 0.07±0.1a | 40.87±3.1a | 4.27±3.0a | 6.79±5.6a | 0.41±0.2a | 8.47±1.5a | 0.53±0.1a |
|  | **7°C** | 21.97±0.9a | 17.28±1.3a | 5.51±0.1a | 20.78±0.8a | 0.78±0.1a | 0.12±0.0a | 36.77±3.5a | 7.87±3.9a | 11.22±5.3a | 0.42±0.1a | 6.60±1.0a | 0.45±0.3a |
| **BC** | **fresh** | 14.44±0.8a | 17.88±0.3a | 5.72±0.1a | 23.97±0.5a | 0.75±0.0a | 0.14±0.1a | 27.36±5.8b | 6.99±4.1a | 13.54±7.4a | 0.33±0.1a | 3.54±1.0a | 0.88±0.1a |
|  | **20°C** | 14.41±0.5a | 18.02±0.2a | 5.42±0.1b | 23.74±0.4a | 0.63±0.2a | 0.09±0.0a | 42.20±2.7a | 4.37±3.2a | 8.58±6.9a | 0.25±0.1a | 6.68±2.4a | 0.83±0.5a |
|  | **7°C** | 13.92±0.6a | 17.74±0.2a | 5.50±0.1b | 24.41±0.8a | 0.74±0.0a | 0.11±0.1a | 35.45±3.6ab | 5.39±4.2a | 10.38±9.2a | 0.35±0.1a | 6.92±1.7a | 0.77±0.2a |
| **BCxR** | **fresh** | 20.36±1.7a | 17.91±0.4a | 6.16±0.1a | 23.59±0.8a | 0.85±0.0a | 0.14±0.1a | 18.41±4.3a | 5.67±5.4a | 7.60±6.0a | 0.39±0.3a | 7.98±3.2a | 0.46±0.0a |
|  | **20°C** | 20.61±1.1a | 17.74±0.7a | 5.81±0.4a | 23.75±1.4a | 0.77±0.0b | 0.08±0.1a | 27.02±10.6a | 4.43±5.0a | 4.53±6.1a | 0.25±0.2a | 10.05±4.1a | 0.81±0.6a |
|  | **7°C** | 20.24±1.1a | 17.03±0.8a | 5.89±0.2a | 25.35±1.1a | 0.79±0.0ab | 0.19±0.1a | 17.68±2.0a | 10.79±6.0a | 10.34±5.2a | 0.40±0.2a | 9.45±2.8a | 0.35±0.2a |
| **R** | **fresh** | 33.01±0.3a | 17.85±1.4a | 6.29±0.3a | 21.04±2.4a | 1.03±0.2a | 0.06±0.0a | 19.19±6.4a | 4.16±3.7a | 5.54±5.1a | 0.26±0.2a | 7.72±2.4a | 0.55±0.2a |
|  | **20°C** | 32.13±0.7a | 17.17±0.9a | 6.33±0.5a | 20.53±0.4a | 0.92±0.1a | 0.04±0.0a | 18.47±7.0a | 2.51±1.9a | 3.00±2.7a | 0.27±0.2a | 8.67±1.9a | 1.07±0.5a |
|  | **7°C** | 32.95±1.1a | 17.20±0.4a | 6.49±0.1a | 21.05±0.3a | 0.93±0.0a | 0.04±0.0a | 10.57±5.5a | 2.17±1.6a | 2.49±2.6a | 0.20±0.1a | 10.64±2.8a | 0.86±0.3a |
| **C/Bl** |  | *** | *** | *** | *** | *** | ** | *** | ns | ns | ns | ** | * |
| **SR** |  | ns | ns | ns | ns | ns | ns | *** | ns | ns | ns | ** | ns |
| **C/Bl x SR** |  | ns | ns | ns | ns | ns | ns | * | ns | ns | ns | ns | ns |

ns = not significant, * = p<0.05, ** = p<0.01, *** = p<0.001

| **C/Bl** | **SR** | **(Z)-3-Hexenol**  **[rel. conc. %]** | **2-Isobutyl**  **thiazole**  **[rel. conc. %]** | **Benzaldehyde**  **[rel. conc. %]** | **Phenylacet**  **aldehyde**  **[rel. conc. %]** | **Neral**  **[rel. conc. %]** | **Geranial**  **[rel. conc. %]** | **Methyl salicylate**  **[rel. conc. %]** | **ß-Damas**  **cenone**  **[rel. conc. %]** | **(E)-Geranyl**  **Acetone**  **[rel. conc. %]** | **2-Phenyl**  **ethanol**  **[rel. conc. %]** | **ß-Ionone**  **[rel. conc. %]** |
| --- | --- | --- | --- | --- | --- | --- | --- | --- | --- | --- | --- | --- |
| **P** | **fresh** | 3.03±1.0a | 6.60±1.5b | 0.29±0.0a | 0.16±0.1a | 0.67±0.2a | 1.53±0.6a | 0.62±0.1b | 1.12±0.4a | 3.39±1.5a | 0.85±0.5a | 1.58±0.9a |
|  | **20°C** | 1.59±0.4b | 9.00±1.3ab | 0.11±0.0b | 0.19±0.0a | 1.07±0.5a | 3.06±1.8a | 0.65±0.2ab | 0.63±0.3a | 3.92±1.1a | 0.56±0.2a | 1.53±0.5a |
|  | **7°C** | 2.04±0.5ab | 9.44±1.0a | 0.15±0.0b | 0.20±0.0a | 1.10±0.1a | 2.85±0.5a | 0.99±0.2a | 0.80±0.2a | 4.13±0.8a | 0.58±0.1a | 1.76±0.5a |
| **BCxP** | **fresh** | 1.27±0.2a | 2.59±0.5a | 0.35±0.2a | 0.17±0.1b | 1.12±0.4a | 2.51±0.8a | 0.67±0.2a | 1.03±0.2a | 2.64±0.8a | 1.87±0.5a | 1.03±0.2a |
|  | **20°C** | 0.62±0.2b | 2.77±0.3a | 0.11±0.0a | 0.46±0.1a | 1.24±0.3a | 2.92±0.5a | 0.36±0.1a | 0.54±0.1b | 3.05±0.5a | 2.17±0.3a | 0.56±0.1b |
|  | **7°C** | 0.70±0.5ab | 2.46±1.0a | 0.11±0.1a | 0.27±0.1ab | 1.03±0.1a | 2.19±0.4a | 0.43±0.3a | 0.51±0.2b | 2.37±0.4a | 1.52±0.4a | 0.54±0.1b |
| **BC** | **fresh** | 1.78±0.2a | 4.40±1.1a | 0.29±0.1a | 0.16±0.0a | 0.73±0.2a | 1.47±0.6a | 0.29±0.1a | 1.01±0.3a | 1.48±0.2a | 0.56±0.2a | 0.58±0.2a |
|  | **20°C** | 0.94±0.7a | 3.47±0.7a | 0.09±0.0b | 0.25±0.1a | 1.05±0.2a | 2.50±0.7a | 0.17±0.1ab | 0.82±0.4a | 1.88±0.4a | 0.48±0.1a | 0.54±0.1a |
|  | **7°C** | 1.21±0.4a | 3.65±1.6a | 0.10±0.0b | 0.35±0.2a | 1.05±0.1a | 2.34±0.3a | 0.14±0.0b | 0.83±0.2a | 1.68±0.5a | 0.32±0.1a | 0.55±0.1a |
| **BCxR** | **fresh** | 1.40±0.2a | 8.31±1.9a | 0.07±0.0a | 0.29±0.1a | 1.30±0.4a | 3.29±1.3a | 0.54±0.1a | 0.74±0.3a | 3.11±0.4ab | 0.78±0.2a | 0.91±0.1a |
|  | **20°C** | 1.39±1.1a | 6.38±1.7a | 0.03±0.0b | 0.18±0.0ab | 1.20±0.5a | 3.58±1.5a | 0.79±0.9a | 0.73±0.3a | 4.21±1.0a | 0.87±0.6a | 1.03±0.1a |
|  | **7°C** | 0.99±0.5a | 9.72±1.6a | 0.04±0.0ab | 0.14±0.0b | 1.19±0.2a | 2.81±0.6a | 0.41±0.3a | 0.74±0.3a | 2.77±0.5b | 0.68±0.5a | 0.72±0.2a |
| **R** | **fresh** | 1.06±0.2a | 21.14±4.1b | 0.05±0.0a | 0.26±0.1a | 1.24±0.3a | 3.27±1.1a | 0.04±0.0a | 0.69±0.1a | 2.95±0.8a | 0.33±0.0a | 1.07±0.3a |
|  | **20°C** | 1.04±0.5a | 28.45±5.1ab | 0.03±0.0a | 0.18±0.1a | 1.13±0.2a | 3.37±0.6a | 0.04±0.0a | 0.49±0.1a | 3.20±0.5a | 0.48±0.1a | 0.97±0.1a |
|  | **7°C** | 1.51±0.7a | 30.86±2.7a | 0.05±0.0a | 0.17±0.1a | 1.06±0.2a | 2.65±0.8a | 0.05±0.0a | 0.66±0.1a | 2.62±0.9a | 0.43±0.1a | 0.95±0.2a |
| **C/Bl** |  | *** | *** | *** | * | ns | * | *** | ns | *** | *** | *** |
| **SR** |  | *** | ** | *** | ns | ns | ns | ns | ** | * | ns | ns |
| **C/Bl x SR** |  | ** | *** | ** | ** | ns | ns | ns | ns | ns | ns | ns |

ns = not significant, * = p<0.05, ** = p<0.01, *** = p<0.001

**Table S2.** Fruit quality parameters (mean ± standard deviation) of the five cultivars/breeding lines with n=4. Different letters indicate significant differences between cultivar/breeding line regardless of fruits stored fresh, at 20°C or at 7°C. Different letters indicate significant differences between the cultivars/breeding lines (Tukey-Test p ≤ 0.05). MHO = 6-Methyl-5-hepten-3-one.

| **Quality parameters** | **P** | **BCxP** | **BC** | **BCxR** | **R** |
| --- | --- | --- | --- | --- | --- |
| **Lycopene [µg/g FW]** | 135.33±39.58A | 123.78±31.10AB | 90.98±26.28B | 132.41±41.43A | 113.37±19.51AB |
| **ß-Carotene [µg/g FW]** | 13.39±2.51A | 9.31±1.45B | 7.63±0.82BC | 6.32±0.87C | 6.75±1.23C |
| **Dry matter [%]** | 7.45±0.34C | 8.59±0.30B | 9.09±0.20A | 6.64±0.26D | 6.33±0.38D |
| **Total soluble solids [°Brix]** | 5.97±0.33B | 7.30±0.39A | 7.53±0.24A | 5.58±0.26C | 5.26±0.21C |
| **Titratable acids [%]** | 0.39±0.05B | 0.47±0.04A | 0.44±0.04A | 0.31±0.03C | 0.30±0.02C |
| **TSS/TA-Ratio** | 15.61±2.27B | 15.58±1.51B | 17.20±1.94AB | 18.21±2.00A | 17.76±1.42A |
| **Fructose [mg/g FW]** | 21.37±2.08B | 27.64±2.60A | 27.95±1.71A | 20.93±1.88B | 18.25±2.02C |
| **Glucose [mg/g FW]** | 16.39±1.79B | 22.11±2.32A | 23.93±1.59A | 16.90±1.58B | 15.49±1.72B |
| **Citric acid [mg/g FW]** | 4.38±0.92B | 5.75±0.99A | 4.54±0.59B | 3.62±0.74B | 3.96±1.13B |
| **Malic acid [mg/g FW]** | 1.01±0.24BC | 1.10±0.26AB | 0.75±0.22C | 0.96±0.21BC | 1.39±0.49A |
| **Potassium [mg/g FW]** | 1.99±0.18B | 2.32±0.21A | 2.52±0.20A | 1.81±0.21BC | 1.73±0.14C |
| **Phosphorus [mg/g FW]** | 0.23±0.05B | 0.27±0.03A | 0.29±0.03A | 0.20±0.01B | 0.20±0.01B |
| **Magnesium [mg/g FW]** | 0.11±0.01B | 0.12±0.01A | 0.13±0.01A | 0.09±0.01C | 0.09±0.00C |
| **Texture [N]** | 7.13±2.14C | 10.94±2.82B | 10.47±2.37B | 9.75±2.06BC | 14.40±3.18A |
| **L*- value** | 51.17±1.07B | 49.12±0.80C | 48.84±0.63C | 52.58±1.75A | 52.69±0.85A |
| **a*- value** | 15.50±1.38A | 5.91±1.44C | 1.22±0.84D | 9.53±2.01B | 16.95±1.03A |
| **b*- value** | 25.02±1.43B | 21.17±0.92C | 14.05±0.62E | 17.81±1.15D | 27.89±1.05A |
| **Chroma** | 29.50±1.30B | 22.16±0.97C | 14.26±0.64E | 20.40±1.21D | 32.70±0.80A |
| **Hue-angle [°]** | 58.20±2.83C | 74.76±3.58B | 85.09±3.16A | 62.02±5.51C | 58.75±2.16C |
| **16:00 [rel. conc. %]** | 15.81±0.63B | 17.75±0.96A | 17.88±0.24A | 17.56±0.69A | 17.40±0.97A |
| **18:00 [rel. conc. %]** | 5.52±0.37C | 5.58±0.16C | 5.55±0.16C | 5.95±0.31B | 6.37±0.32A |
| **18:01 [rel. conc. %]** | 20.88±0.39B | 20.82±0.94B | 24.04±0.59A | 24.23±1.31A | 20.87±1.32B |
| **18:02 [rel. conc. %]** | 50.50±0.68A | 47.81±1.46B | 46.64±0.75B | 44.66±1.00C | 47.64±1.46B |
| **18:03 [rel. conc. %]** | 6.04±0.44A | 6.55±0.65A | 4.58±0.29B | 6.39±0.68A | 6.11±0.99A |
| **20:00 [rel. conc. %]** | 0.75±0.06BC | 0.83±0.06B | 0.71±0.11C | 0.80±0.05BC | 0.96±0.13A |
| **1-Penten-3-one [rel. conc. %]** | 0.07±0.04B | 0.10±0.06AB | 0.11±0.05AB | 0.14±0.10A | 0.05±0.04B |
| **Hexanal [rel. conc. %]** | 23.24±7.01B | 35.05±7.19A | 35.00±7.41A | 21.04±7.53B | 16.08±7.04B |
| **(Z)-3-Hexenal [rel. conc. %]** | 5.41±5.44A | 4.91±3.56A | 5.59±3.66A | 6.96±5.72A | 2.94±2.48A |
| **(E)-2-Hexenal [rel. conc. %]** | 7.45±6.82AB | 8.03±5.81AB | 10.83±7.45A | 7.49±5.79AB | 3.68±3.56B |
| **(E)-2-Heptenal [rel. conc. %]** | 0.23±0.13A | 0.37±0.15A | 0.31±0.12A | 0.35±0.20A | 0.24±0.16A |
| **MHO [rel. conc. %]** | 6.72±3.29AB | 7.45±1.61AB | 5.72±2.29B | 9.16±3.24A | 9.01±2.53A |
| **1-Hexanol [rel. conc. %]** | 0.67±0.22A | 0.49±0.17A | 0.83±0.30A | 0.54±0.41A | 0.83±0.39A |
| **(Z)-3-Hexenol [rel. conc. %]** | 2.22±0.87A | 0.86±0.41B | 1.31±0.55B | 1.26±0.68B | 1.20±0.53B |
| **2-Isobutylthiazole [rel. conc. %]** | 8.34±1.75B | 2.60±0.65C | 3.84±1.16C | 8.14±2.12B | 26.82±5.67A |
| **Benzaldehyde [rel. conc. %]** | 0.18±0.09A | 0.19±0.17A | 0.16±0.11AB | 0.05±0.03BC | 0.04±0.01C |
| **Phenylacetaldehyde [rel. conc. %]** | 0.18±0.06A | 0.30±0.16A | 0.25±0.15A | 0.20±0.09A | 0.20±0.09A |
| **Neral [rel. conc. %]** | 0.95±0.35A | 1.13±0.27A | 0.94±0.24A | 1.23±0.37A | 1.15±0.22A |
| **Geranial [rel. conc. %]** | 2.48±1.25AB | 2.54±0.62AB | 2.10±0.69B | 3.23±1.12A | 3.10±0.83AB |
| **Methyl salicylate [rel. conc. %]** | 0.76±0.24A | 0.49±0.21AB | 0.20±0.09BC | 0.58±0.53A | 0.04±0.01C |
| **ß-Damascenone [rel. conc. %]** | 0.85±0.35A | 0.69±0.29A | 0.89±0.26A | 0.73±0.28A | 0.61±0.13A |
| **(E)-Geranylacetone [rel. conc. %]** | 3.81±1.09A | 2.69±0.62B | 1.68±0.38C | 3.37±0.90AB | 2.92±0.70AB |
| **2-Phenylethanol [rel. conc. %]** | 0.66±0.33B | 1.85±0.45A | 0.45±0.15B | 0.78±0.40B | 0.41±0.09B |
| **ß-Ionone [rel. conc. %]** | 1.62±0.60A | 0.71±0.28BC | 0.56±0.12C | 0.89±0.20BC | 0.99±0.19B |


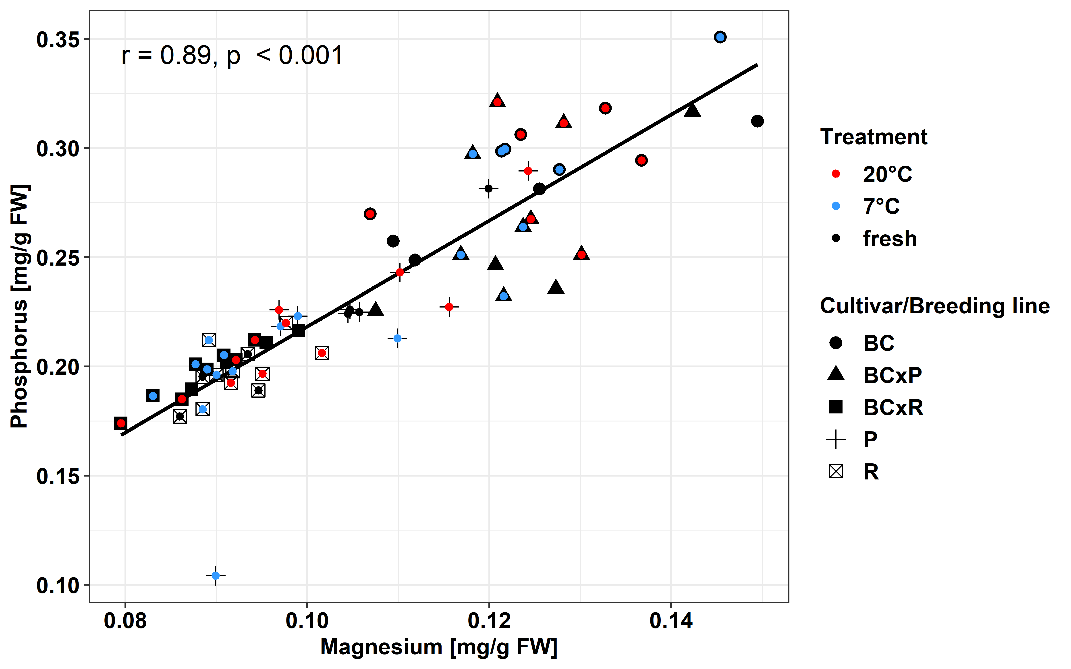


**Figure S1.** Pearson-correlation of magnesium and phosphorous. Values are means of n = 4; correlations were significant with *p* ≤ 0.05.

**Table S3.** Pearson-correlation of carotenoid-derived volatiles and precursors in tomato fruits (n = 60).

|  | **a-value** | **Lycopene** | **ß-Caro-tene** | **6-Methyl-5-hepten-2-one** | **Neral** | **Geranial** | **Trans-Geranyl-acetone** | **ß-Ionone** |
| --- | --- | --- | --- | --- | --- | --- | --- | --- |
| **a-value** | 1 | 0.353^**^ | 0.253 | 0.320^*^ | 0.108 | 0.293^*^ | 0.573^**^ | 0.575^**^ |
| **Lycopene** |  | 1 | 0.327^*^ | 0.261^*^ | 0.082 | 0.169 | 0.448^**^ | 0.213 |
| **ß-Carotene** |  |  | 1 | -0.098 | -0.152 | -0.136 | 0.321^*^ | 0.536^**^ |
| **6-Methyl-5-hepten-2-one** |  |  |  | 1 | 0.731^**^ | 0.811^**^ | 0.502^**^ | 0.239 |
| **Neral** |  |  |  |  | 1 | 0.914^**^ | 0.335^**^ | 0.115 |
| **Geranial** |  |  |  |  |  | 1 | 0.504^**^ | 0.274^*^ |
| **Trans-Geranylacetone** |  |  |  |  |  |  | 1 | 0.786^**^ |
| **ß-Ionone** |  |  |  |  |  |  |  | 1 |

*. The correlation is significant at the level of 0.05 (2-sided).

**. The correlation is significant at the level of 0.01 (2-sided).

**Table S4.** Pearson-correlation of fatty acid-derived volatiles and precursors in tomato fruits (n = 60).

|  | **Linoleic acid (18:2)** | **Linolenic acid (18:3)** | **Hexa-**  **nal** | **(Z)-3-Hexe-nal** | **(E)-2-Hexe-nal** | **(E)-2-Heptenal** | **Hexa-**  **nol** | **(Z)-3-Hexenol** |
| --- | --- | --- | --- | --- | --- | --- | --- | --- |
| **Linoleic acid (18:2)** | 1 | -0.090 | 0.043 | -0.122 | -0.091 | -0.207 | 0.130 | 0.329^*^ |
| **Linolenic acid (18:3)** |  | 1 | -0.292^*^ | -0.066 | -0.207 | -0.074 | -0.332^**^ | -0.092 |
| **Hexanal** |  |  | 1 | -0.072 | 0.120 | 0.132 | -0.121 | -0.449^**^ |
| **(Z)-3-Hexenal** |  |  |  | 1 | 0.865^**^ | 0.679^**^ | -0.461^**^ | -0.183 |
|  |  |  |  |  |  |  |  |  |
| **(E)-2-Hexenal** |  |  |  |  | 1 | 0.747^**^ | -0.401^**^ | -0.162 |
| **(E)-2-Heptenal** |  |  |  |  |  | 1 | -0.284^*^ | -0.290^*^ |
| **Hexanol** |  |  |  |  |  |  | 1 | 0.508^**^ |
| **(Z)-3-Hexenol** |  |  |  |  |  |  |  | 1 |

*. The correlation is significant at the level of 0.05 (2-sided).

**. The correlation is significant at the level of 0.01 (2-sided).

**Table S5.** Sensory results (mean ± standard deviation) of the five cultivars/breeding lines for fresh harvested fruits (fresh), after 20°C and 7°C household storage and sensory results (mean ± standard deviation) of the five cultivars/breeding lines regardless of the treatment. Different letters indicate significant differences between fresh harvested fruits, after 20°C and 7°C household storage for each cultivar/breeding line (Tukey-Test *p* ≤ 0.05) and indicate differences between the cultivars/breeding lines regardless of the treatment (Tukey-Test *p* ≤ 0.05). A two-way ANOVA was performed as well (C = Cultivar, Bl = Breeding line, SR = Storage regime).

| **C/Bl** | **SR** | **Green/Grassy Odor** | **Tomato-like Odor** | | **Tomato-like Flavor** | | **Sweetness** | | **Sourness** | | **Juiciness** | | **Skin Strength** | | **Aftertaste** | |
| --- | --- | --- | --- | --- | --- | --- | --- | --- | --- | --- | --- | --- | --- | --- | --- | --- |
| **P** | **Fresh** | 44.03 ± 8.96a | 43.23 ± 5.81a | | 41.21 ± 5.82a | | 51.91 ± 12.74a | | 29.03 ± 6.69a | | 42.65 ± 8.55a | | 54.4 ± 13.03a | | 33.9 ± 9.33a | |
|  | **20°C** | 46.55 ± 14.12a | 39.22 ± 6.94a | | 38.85 ± 6.02a | | 47.77 ± 12.97a | | 29.99 ± 10.45a | | 48.15 ± 12.46a | | 52.4 ± 15.09a | | 33.02 ± 9.23a | |
|  | **7°C** | 48.24 ± 10.65a | 42.06 ± 9.40a | | 40.06 ± 8.14a | | 47.32 ±10.82a | | 31.73 ± 9.94a | | 43.63 ± 6.86a | | 55.95 ± 10.60a | | 33.23 ± 9.97a | |
| **BCxP** | **Fresh** | 43.19 ±7.08a | 39.39 ± 5.40a | | 41.94 ± 8.93a | | 59.99 ± 12.94a | | 35.14 ± 8.59a | | 39.28 ± 9.59a | | 61.79 ± 8.75a | | 41.94 ± 8.83a | |
|  | **20°C** | 44.54 ± 9.85a | 38.04 ± 9.45a | | 45.65 ± 13.13a | | 61.53 ± 18.17a | | 30.95 ± 9.54a | | 40.75 ± 7.61a | | 59.88 ± 10.76a | | 34.86 ± 12.54a | |
|  | **7°C** | 44.82 ± 7.30a | 38.99 ± 11.15a | | 45.65 ± 13.28a | | 61.68 ± 21.79a | | 29.47 ± 9.94a | | 43.78 ± 10.65a | | 57.74 ± 14.22a | | 35.48 ± 14.32a | |
| **BC** | **Fresh** | 42.11 ± 9.12a | 37.9 ± 5.70a | | 41.2 ± 9.11a | | 59.49 ± 14.74a | | **31.36 ± 8.16ab** | | 43.98 ± 12.37a | | 58.4 ± 15.82a | | 39.78 ± 9.08a | |
|  | **20°C** | 44.75 ± 8.20a | 36.38 ± 10.10a | | 42.54 ± 13.25a | | 56.45 ± 19.70a | | **33.98 ± 7.49a** | | 44.45 ± 11.61a | | 58.43 ± 14.23a | | 35.92 ± 13.72a | |
|  | **7°C** | 42.88 ± 11.48a | 37.25 ± 8.85a | | 43.73 ± 15.24a | | 65.18 ± 18.50a | | **27.48 ± 10.67a** | | 45.31 ± 7.47a | | 56.00 ± 14.27a | | 35.85 ± 12.74a | |
| **BCxR** | **Fresh** | 45.25 ± 8.39a | 38.04 ± 5.98a | | 36.82 ± 8.20a | | 49.87 ± 11.04a | | 30.35 ± 8.50a | | 43.28 ± 12.42a | | 57.97 ± 8.96a | | 30.25 ± 8.25a | |
|  | **20°C** | 40.45 ± 11.40a | 38.65 ± 4.71a | | 36.77 ± 7.49a | | 50.09 ± 14.14a | | 25.26 ± 9.50a | | 35.22 ± 9.43a | | 56.88 ± 12.45a | | 32.34 ± 9.04a | |
|  | **7°C** | 39.52 ± 12.13a | 33.96 ± 8.49a | | 34.34 ± 10.17a | | 44.56 ± 9.94a | | 27.56 ± 7.63a | | 39.54 ± 13.51a | | 52.66 ± 12.10a | | 29.08 ± 9.70a | |
| **R** | **Fresh** | 39.69 ± 11.83a | **33.6 ± 9.30b** | | 36.45 ± 7.25a | | 45.1 ± 9.85a | | 28.63 ± 6.61a | | 40.38 ± 12.83a | | 59.81 ± 9.64a | | 28.8 ± 9.04a | |
|  | **20°C** | 41.15 ± 12.72a | **39.67 ± 6.77a** | | 36.51 ± 5.38a | | 42.14 ± 11.62a | | 29.91 ± 9.60a | | 35.03 ± 10.47a | | 55.63 ± 11.29a | | 28.84 ± 7.33a | |
|  | **7°C** | 40.08 ± 13.86a | **36.8 ± 5.60ab** | | 34.75 ± 6.58a | | 39.04 ± 14.08a | | 27.45 ± 9.27a | | 38.09 ± 9.56a | | 56.7 ± 8.25a | | 24.53 ± 8.54a | |
| **C/Bl** |  | * | ** |  | *** |  | *** |  | ns |  | *** |  | ns |  | *** |  |
| **SR** |  | ns | ns |  | ns |  | ns |  | ns |  | ns |  | ns |  | ns |  |
| **C/Bl x SR** |  | ns | ns |  | ns |  | ns |  | ns |  | ns |  | ns |  | ns |  |
| **P** |  | 46.41 ± 11.53a | 41.40 ± 7.70a |  | 39.97 ± 6.76ab |  | 48.83 ± 12.16b |  | 30.33 ± 9.24a |  | 44.94 ± 9.80a |  | 54.30 ± 12.93a |  | 33.35 ± 9.39ab |  |
| **BCxP** |  | 44.24 ± 8.14ab | 38.77 ± 9.05ab |  | 44.56 ± 12.05a |  | 61.13 ± 18.01a |  | 31.66 ± 9.47a |  | 41.39 ± 9.40ab |  | 59.69 ± 11.56a |  | 37.16 ± 12.50a |  |
| **BC** |  | 43.31 ± 9.65ab | 37.13 ± 8.45b |  | 42.56 ± 12.83a |  | 60.42 ± 18.07a |  | 30.91± 9.22a |  | 44.61 ± 10.45a |  | 57.56 ± 14.55a |  | 37.03 ± 12.12a |  |
| **BCxR** |  | 41.85 ± 10.82ab | 36.92 ± 6.78b |  | 36.00 ± 8.61b |  | 48.23 ± 11.89b |  | 27.81 ± 8.69a |  | 39.47 ± 12.20b |  | 55.90 ± 13.13a |  | 30.54 ± 8.94bc |  |
| **R** |  | 40.29 ± 12.60b | 36.59 ± 7.76b |  | 35.92 ± 6.42b |  | 42.19 ± 11.98b |  | 28.66 ± 8.46a |  | 37.91 ± 11.15b |  | 57.46 ± 9.80a |  | 27.43 ± 8.47c |  |

ns = not significant, * = p<0.05, ** = p<0.01, *** = p<0.001

**Figure S2.** Sensory evaluation of five different cultivars/breeding lines. Spider webs (**A-E**) show the results for each cultivar/breeding line, with the lines representing either fresh fruits, fruits stored at 20°C, or fruits stored at 7°C household storage.
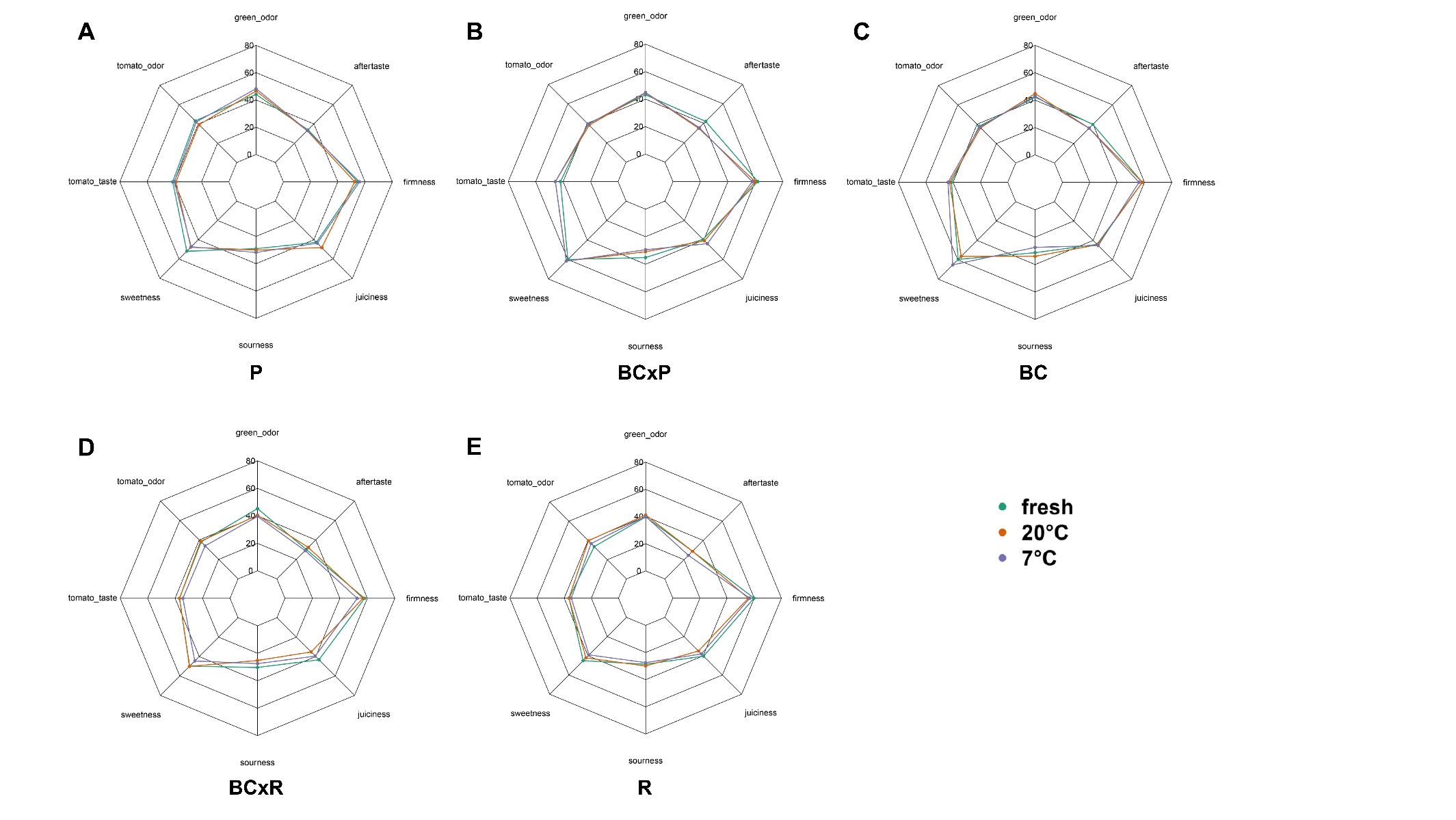

Supplement: Supplementary file 1 [file Data_Sheet_1.docx]
